# Exploring conserved mRNA-miRNA interactions in colon and lung cancers 

**Published:** 2017

**Authors:** Fereshteh Izadi, Mona Zamanian-Azodi, Vahid Mansouri, Mahsa Khodadoostan, Nosratollah Naderi

**Affiliations:** 1 *Gastroenterology and Liver Diseases Research Center, Research Institute for Gastroenterology and Liver Diseases, Shahid Beheshti University of Medical Sciences, Tehran, Iran *; 2 *Proteomics Research Center, Shahid Beheshti University of Medical Sciences, Tehran, Iran*; 3 *Physiotherapy Research Center, Shahid Beheshti University of Medical Sciences, Tehran, Iran*; 4 *Department of Gastroenterology and Hepatology, Isfahan University of Medical Sciences, Isfahan, Iran*; 5 *Basic and Molecular Epidemiology of Gastrointestinal Disorders Research Center, Research Institute for Gastroenterology and Liver Diseases, Shahid Beheshti University of Medical Sciences, Tehran, Iran*

**Keywords:** Colon cancer, Gene regulatory network, Lung cancer, miRNA

## Abstract

**Aim:**

The main goal of this analysis was prioritization of co-expressed genes and miRNAs that are thought to have important influences in the pathogenesis of colon and lung cancers.

**Background:**

MicroRNAs (miRNAs) as small and endogenous noncoding RNAs which regulate gene expression by repressing mRNA translation or decreasing stability of mRNAs; they have proven pivotal roles in different types of cancers. Accumulating evidence indicates the role of miRNAs in a wide range of biological processes from oncogenesis and tumor suppressors to contribution to tumor progression. Colon and lung cancers are frequently encountered challenging types of cancers; therefore, exploring trade-off among underlying biological units such as miRNA with mRNAs will probably lead to identification of promising biomarkers involved in these malignancies.

**Methods:**

Colon cancer and lung cancer expression data were downloaded from Firehose and TCGA databases and varied genes extracted by DCGL software were subjected to build two gene regulatory networks by parmigene R package. Afterwards, a network-driven integrative analysis was performed to explore prognosticates genes, miRNAs and underlying pathways.

**Results:**

A total of 192 differentially expressed miRNAs and their target genes within gene regulatory networks were derived by ARACNE algorithm. BTF3, TP53, MYC, CALR, NEM2, miR-29b-3p and miR-145 were identified as bottleneck nodes and enriched via biological gene ontology (GO) terms and pathways chiefly in biosynthesis and signaling pathways by further screening.

**Conclusion:**

Our study uncovered correlated alterations in gene expression that may relate with colon and lung cancers and highlighted the potent common biomarker candidates for the two diseases.

## Introduction

 The complex molecular interactions underlying cancers warrant identification of biological entities like miRNAs as well as the crosstalk between different cancers. Colon cancer is a fatal malignancy with estimated 1.4 million cases yearly (1). Despite much research to elucidate the molecular processes that promote the normal colon cells toward tumors, the rate and average years of survival have not changed considerably over decades. Lung cancer is also a worldwide fatal cancer with high prevalence even at early stage (2) that can be grouped in two major forms; non-small cell lung cancer (NSCLC ≈85% of all lung cancers) and small-cell lung cancer (SCLC ≈15%). Furthermore, NSCLC can be divided into three major histological sub-types: squamous-cell carcinoma, adenocarcinoma and large-cell lung cancer (3). As both colon and lung tissues derive from primordial gut, supposedly the same genes may be involved in regulatory networks underlying cellular pathways in the two organs. Therefore, tumorigenesis in both of these organs may also be partly similar by implicating the same sets of genes and regulatory units. Reportedly, the majority of genes involved in colon and lung cancers are likely identical or related (4). Moreover, experimental evidence showed the role of a number of genes including LRRC36 (5), SLC6A4 (6), COL3A1 (7), TMEM125 (8), ITGAV (9), TXNDC17 (10) and PPP2R5A (11) in both colon and lung cancers that may imply a possible correlation between the two malignancies at transcriptome level (12). Nowadays, data mining approaches including networking constitute a prominent strategy for extracting meaningful information from a growing wealth of biological data such as gene expression profiles (13). Biological networks are mostly inferred by transcriptomics profiles including microarrays and next generation sequencing (NGS) platforms, and microarrays have been used intensively (14). Rapid advances in NGS techniques have deviated the massive employment of microarrays as the main expression platform to sequencing data (15). In addition to lower sensitivity because of unavoidable noise coming from the nature of microarrays, NGS techniques offers several advantages over microarrays (14, 16). These techniques do not depend on genomic pre-knowledge for transcriptome analysis and can be utilized for model and non-model organisms (17). While microarrays can cover only the characterized parts of genome, NGS platforms are able to identify entire transcripts (18). Finally, detecting the novel transcripts and variant splicing are other capabilities of NGS profiling techniques (19, 20). In this study, it was hypothesized that there is a correlation between mRNAs and miRNAs in colon and lung cancers at transcriptional level. Therefore, network mining was conducted to identify putative miRNA-mRNA modules in these cancers. 

## Methods

**Data acquisition and pre-processing**

In the present article, colon cancer gene expression dataset generated by Illuminaga_RNASeqV2 containing 192 normal and cancer samples and 20532 genes was chosen from Firehose (https://confluence.broadinstitute.org/display/GDAC/Home). For the miRNA expression profiling data, lung cancer Illumina HiSeq included 231 normal and cancer samples and 1045 genes were retrieved from TCGA (https://www.synapse.org/#!Synapse:syn300013/wiki/27406). The expression values of miRNAs and mRNAs were subjected to expression Based filter and variance Based filter functions implemented in DCGL v2.0 R package (21) to filter out genes that are extremely expressed invariably between normal and cancer samples. The expression Based filter removes genes whose mean expression between experiments is lower than the median of this value for all genes and the variance Based filter removes genes that are expressed not significantly more variably than the median gene. 

**In silico analysis and networking**

Predicted gene-miRNA interactions were collected from miRWalk 2.0 server (http://zmf.umm.uni-heidelberg.de/apps/zmf/mirwalk2/miRpub.html) based on miRBase database. Regarding the mRNAs, only the miRNAs target genes were retrieved by miRWalk from colon cancer transcripts. Afterwards, two independent gene regulatory networks were built using unique miRNAs and their target genes using arcana function with eps=0.05 implemented in parmigene R package (22). Next, genes and miRNAs within the networks were separately ranked based on betweenness centrality as bottlenecks measure by CytoNCA Cytoscape plugin (23). Moreover, interacting transcription factors among the bottleneck mRNAs were extracted by TransmiR (http://www.cuilab.cn/transmir), a database of experiment-supported TF–miRNA regulatory interactions manually curated from publications. Finally, the identified gene-miRNA-TF modules were plotted using Cytoscape 3.4.0 (24). 

**Gene Ontology analysis and visualization**

To find the significantly over-represented biological GO terms and functions of gene products within regulatory network mRNAs, functional classification was performed using BINGO Cytoscape plugin (25) running hypergeometric test and Benjamini & Hochberg with False Discover Rate (FDR) correction at a significance level of 0.01. Finally, the clusters were visualized by Enrichment map Cytoscape plugin with Jaccard’s coefficient 0.001. mRNAs were further functionally classified by PANTHER database (http://pantherdb.org/) to underlying pathways (Figure 4).

In order to study the functional roles of miRNA and their targets in biological pathways, pathway analysis was performed using PANTHER (http://www.pantherdb.org/geneListAnalysis.do) and mirPath v.3 (http://snf-515788.vm.okeanos.grnet.gr/), respectively. 

## Results

**Building miRNA-mRNA interaction networks**

**Figure 1:**
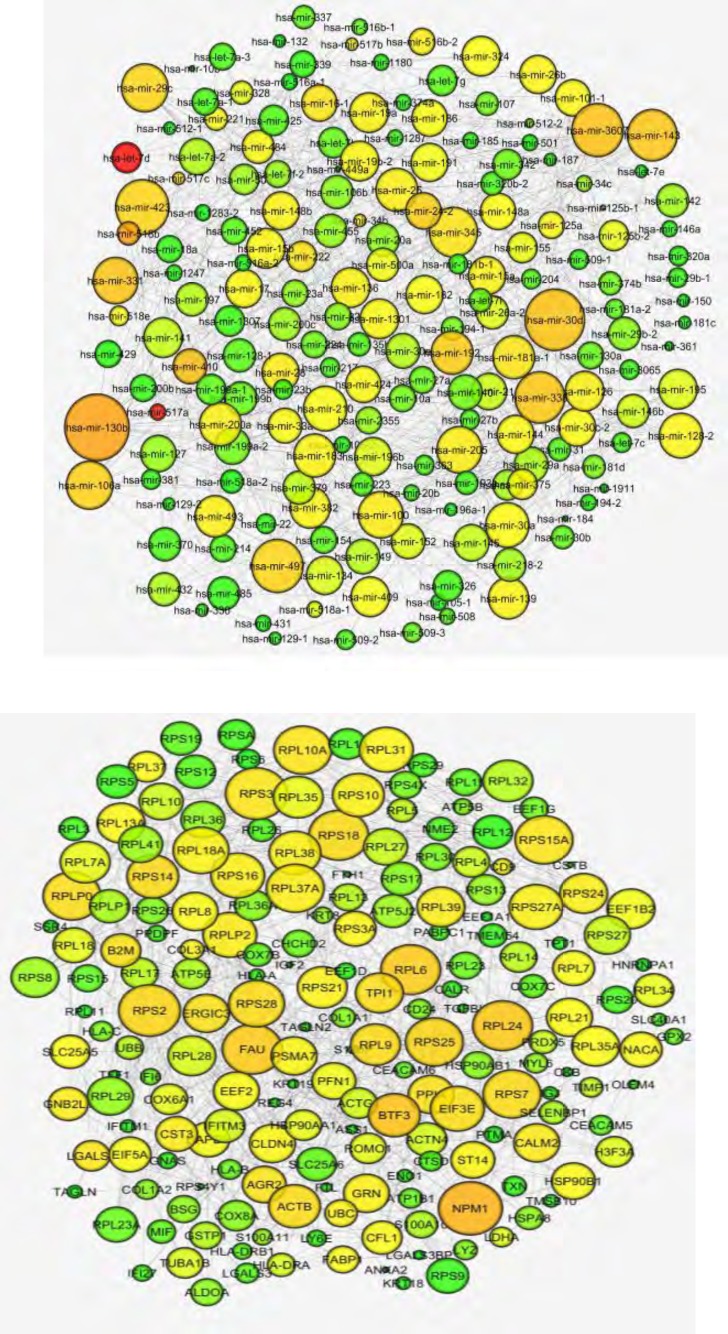
Lung (upper) and colon (lower) interaction networks inferred by ARACNE algorithm. Using NetworkAnalyzer Cytoscape plugin we mapped degree and betweeness parameters to node size and color intensity respectively so that bigger and darker nodes show higher degree and betweeness centralities

The 192 most variable miRNAs and target transcripts extracted from lung and colon cancer data were subjected to build two independent interaction networks by ARACNE algorithm implemented in parmigene software (setting epsilon to 0.05). Information-theoretic approaches like ARACNE (20) have been successfully applied for inferring large networks (26). In these approaches, first a pair-wise mutual information (MI) matrix is calculated between all possible pairs of genes. Afterwards, this matrix is manipulated to identify regulatory interactions between nodes (15). For more convenience in visualizing the networks in Cytoscape, we only selected the first 1000 highly ranked edges. By utilizing the Network Analyzer (27), nodes within networks with higher connections were set to darker color and bigger size (Figure 1). 

As shown in Figure 1, miR-1283-2, miR-129-1, let-7 and miR-424 showed the highest connections within miRNA interaction network. Regarding colon cancer network, genes including NME2, ATP1A1, CD24 and IFI6 showed the most connectivity. 

**Exploring miRNA–TF–mRNA network**

**Figure 2:**
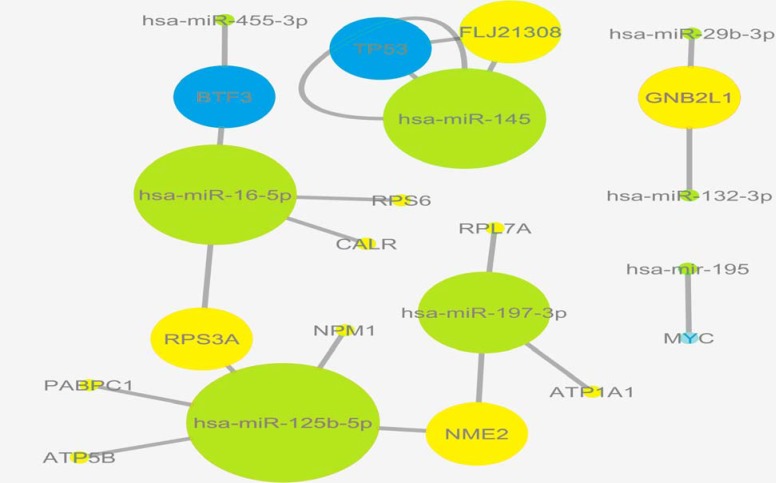
miRNA–TF–gene interaction network. Green circles donate miRNAs, yellows denote target genes of miRNAs, and blues denote TFs targets of miRNAs in colon and lung cancers

The 192 miRNA variable transcripts of lung cancer data targeted 13711 validated genes based on miRWalk server. We then intersected between these 13711 targets and the most variable mRNAs in colon cancer data extracted from DCGL R package. As the parmigene method demands orthogonal matrices for inferring co-expression network, we then processed further with two matrices of miRNAs and mRNAs both including 192 samples and genes. Degree distribution and following motif discovery (28) and recently betweenness centrality (29) are some of topological features that can address vulnerable points within biological networks. Next, for exploring putative miRNA-TF-mRNA networks underlying colon and lung cancers, we focused on the genes and miRNAs with the highest betweenness centrality within networks. To do so, we analyzed the upstream of 192 target genes with iRegulon Cytoscape plugin (30). By intersecting iRegulon results and 10% of 192 targets with the highest betweenness centrality, we identified a list of TFs among the targets. As shown in Figure 2, we identified BTF3, TP53 and MYC TFs as interacting TFs with miR-455-3p, miR-144 and miR-195 among 10% of lung cancer miRNAs with the highest betweenness. 

**Figure 3:**
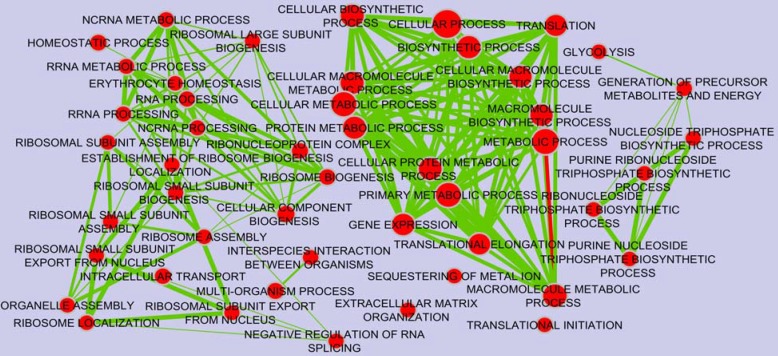
Functional classification of biological processes in which differential expressed genes from colon cancer supposed to be involved. The GO terms considered significant based on hypergeometric test with Benjamini & Hochberg FDR correction and significance level 0.01 by BINGO app. The results was illustrated using Enrichment map Cytoscape plugin. The bigger red circles and ticker green lines show GO terms that contain more genes with higher significance level

The crossroads among the screened miRNAs, genes and TFs was illustrated in Figure 2 as modules, detonating each unit with more connection in bigger size. 

**Figure 4:**
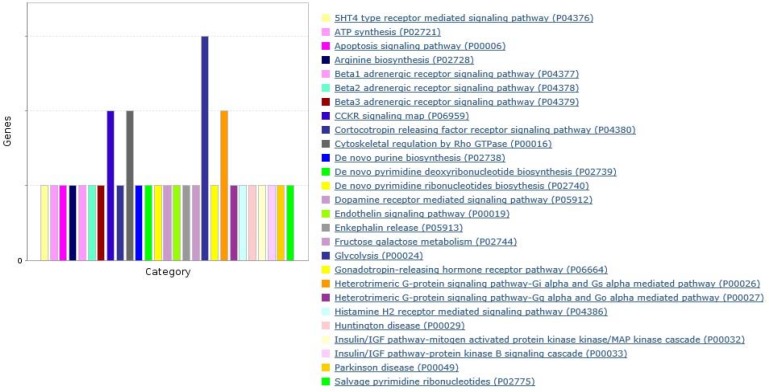
Bar chart of biological pathways in which variable transcripts extracted from colon cancer data potentially involved by PANTHER server with default parameters. The length of y axis showes the number of genrs assigned to each pathway

**Figure 5:**
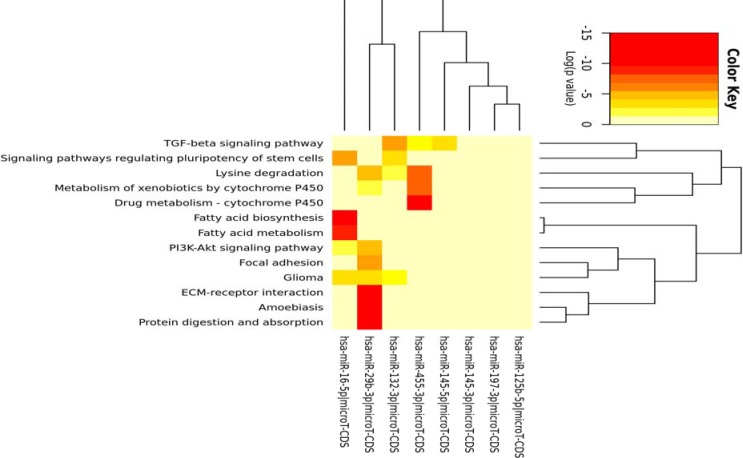
Heatmap of KEGG pathways in which bottleneck miRNAs after FDR correction were enriched. miRNAs were uploaded in mirPath v.3 server at p-value < 0.01

**GO and pathway functional enrichment analysis**

As shown in Figures 3 and 4, 192 colon cancer related genes as validated targets of lung cancer miRNAs, were enriched in biological GO terms and pathways including glycolysis, purine and arginine biosynthesis, extra-cellular matrix organization, sequestering of metal ion and apoptosis as well as signaling pathways like endothelin and G-protein among which the representation of several of biosynthetic processes were more noteworthy. Furthermore, mirPath v.3 at p-value 0.01 threshold after FDR correction was applied to investigate the KEGG pathways in which bottleneck miRNAs are likely implicated. Heatmap denotes union of significant pathways. As shown in Figure 5, signaling pathways TGF-beta, Pl3K-Atk and pluripotency of stem cells as well as fatty acid metabolism have been reported to be involved in colon and lung cancers (31-34). 

## Discussion

By representing the molecular interactions underlying biological processes, network biology paves the path to drug discovery, better understanding of diseases mechanism and cancer therapeutics (35-37). miRNAs, mostly 19 to 25 nucleotide-long non-coding RNAs, biological units with low complexity, high stability and easy detection, regulate gene expression at the level of mRNA degradation and translation (38). Due to the fact that miRNAs play fundamental roles in development, differentiation and involvement of biological mechanisms underlying tumorigenesis, they could be promising biomarkers for several types of cancers. Cancer-related mortality rates in colon and lung malignancies is still the most common causes of cancer death worldwide; therefore, detecting biomarkers of initiation and progression of these cancers are challenging topics in cancer biology (1). In graph theory, in addition to hub nodes (proteins with high degrees), bottleneck nodes with higher betweenness centrality (a function of shortest paths that pass through a node) are more likely to be key connectors and likewise critical points controlling important dynamic components in biological networks (39). Removing them causes biological systems failure to save their coherence; therefore, these nodes were named bottleneck nodes (40, 41). In this article, open NGS data and bioinformatics tools were employed to explore the miRNAs and genes that are expressed in colon and lung tissues whose relationships hypothetically propel these tissues toward cancer. Two independent gene regulatory networks were constructed using the differentially expressed miRNAs and mRNAs from lung and colon cancers data with a novel mutual information estimation method named parmigene. The networks were then topologically analyzed to find the bottleneck miRNAs and genes. Bottleneck miRNAs and mRNAs were highlighted if they were in the top 10% of degree distribution (genes that have the 10% highest number of neighbors) within the inferred miRNAs and genes interaction networks. The top 5% nodes are reported as bottleneck in previous studies; nevertheless, cutoff determination depends on the research profile and is arbitrary (42). It has been shown that selecting genes in range of 10-40% of degree distribution does not have a significant effect on the results (43). Of note, the colon cancer related genes were solely selected among the validated targets of 192 miRNAs. Massive literature search showed that all bottleneck miRNAs and their targets are involved in colon and lung cancers. By the finest explanation, this could be biologically relevant with similarity of these tissues in cancer associated pathways. The identified miRNAs included miR-145 (44, 45), miR-197-3p (46), miR-16-5p (47), miR-455-3p (48), miR-195 (49, 50), miR-132-3p (51) and miR-29b-3p (52) among which miR-132-3p , miR-29b-3p , miR-16-5p and miR-455-3p were enriched in more significant pathways like protein and lysine degradation, focal adhesion and cytochrome P450 (Figure 5), likely to be more involved in metabolism homeostasis and carcinogenesis (53). miR-16-5p is considered as a reference miRNA that was related with fatty and metabolism in this study. Moreover, target genes of miRNAs were enriched in pathways characterized to be associated with cancer, including enkephalin release involved in immune system (54), endothelin (55), a number of signaling pathways like G-protein (56, 57), apoptosis (58, 59) as well as ATP (60), purine (61), arginine biosynthetic processes (62, 63) and glycolysis (64). The relationship between G-protein signaling pathway and fatty acids has been demonstrated in colon cancer (65). Target genes of bottleneck miRNAs were also classified in pathways presumably related with colon cancer, like biosynthetic processes, sequestering ion, extra-cellular matrix organization and erythrocyte homeostasis. Supposedly biosynthetic processes as the most common processes among miRNAs and mRNAs can be fairly explained thus; flux through glycolysis provides biosynthetic processes like purine, arginine, etc., and in the absence of these fuels, cancer cells will die due to apoptotic death. Concordantly, a line of evidence reported the fundamental roles of biosynthesis in cancer progression (66, 67). Additionally, among the bottleneck target genes, NME2, CARL, FLJ1308 and CD24 are more prominent. The expression of NME2 was found to be reduced in different cancers including colon cancer (68). Calreticulin (CALR) was shown to contribute to breast cancer progression by dysregulation of TP53 transcription factor (69). In this study, CALR appeared to be regulated by miR-16-5p that is itself enriched in fatty acid biosynthesis. Finally, to recognize the potential master transcription factors among the target genes furthermore illustrating the modules of TFs-miRNAs-genes, we intersected between iRegulon analysis results, bottleneck targets and a list of interacting TFs with bottleneck miRNAs from TransmiR server. Notably, three transcription factors BTF3, Myc and TP53 were found to be associated with miR-455-3p, miR-195 and miR-145, respectively (Figure 2). BTF3 transcription factor was shown to be overexpressed in colorectal cancer (70); in addition, a number of studies have described the role of TP53 in colorectal cancer (71, 72). Among the target genes, FLJ1308 was detected to interact with both miR-145 and TP53. Apoptotic loops of miR-145 and TP53 in tumor prevention have been recently identified (73-75). Tumor suppressor TP53 regulates a wide range of signals among which, in this analysis, the biosynthesis processes in response to nutrition depletion and representation of MYC oncogene in bottleneck target genes were more interesting. We observed that cancer-related modules of miRNAs-target genes-TFs in both colon and lung cancers are common in several biological pathways, indicating similarity between these tissues regarding cancer progression. 

We aimed to explore biomarkers underlying both colon and lung cancers ;therefore, in the frame of network mining methods and topology feature analysis, a small number of putative genes, miRNAs and transcription factors were explored whose interplays are probably related to colon and lung cancers. An expected outcome of such a work would possibly identify crosstalk of miRNAs and genes in tumorigenesis in different tissues and more evidence for cancer diagnosis and treatment. In the present study, NGS data was chosen over microarray because: I) this technique provides more sensitive detection of transcripts, which is likely to be the reason for the ability of NGS data to detect low expressed genes while microarrays fail to differentiate between very low expressed and non-expressed genes (76); II) accurate measurement of the dynamic range of low and highly expressed genes (77); and III) giving a better resolution of relationship between biological units, especially for profiling of RNA molecules in which short reads should pass from adapter removal filter. 

However, this analysis is challenged by some limitations. Firstly, despite well-demonstrated roles of miRNAs in regulating multiples target genes involved in different oncogenic pathways in cancers, we should be cautious about the fact that each miRNA could potentially target hundreds of genes. Therefore, we need a deeper understanding of miRNA biology and undeniable role of experimental practices to improve fidelity of bioinformatics results. Next, evidently from ontology analysis, target genes could barely provide a clear biological finding; therefore, research works addressing validating miRNA sites within mRNAs will decrease the ambiguity in defining regulatory interactions among miRNAs-targets. Furthermore, we inferred information-theoretic based undirected networks while connectivity between nodes does not mean the causal relationships; we then should be cautious about dynamic nature of cancers via strict analysis of statics networks. Additionally, it is essential to remove overestimated regulation dependencies by employing more sophisticated gene regulatory inference algorithms. Finally, network analysis at transcriptome level could be more intensified through merging studies with protein networks to draw more practical conclusions.

It is concluded that utilizing dual information of miRNAs and mRNAs in cancers trade-off can help to discover important findings to identify underlying mechanisms and enlighten more molecular underpinnings of different cancers. We observed that the identified miRNAs-mRNA covered a wide range of known functions, mainly signaling pathways and biosynthesis implicated in colon and lung cancers.

To summarize, conserved miRNAs and TFs like miR-195, miR-145, BTF3, Myc and TP53 with their targets could be considered as hallmark genes for future diagnosis and therapeutic researches where their rules could be confirmed by experiments.

Utilizing dual information of miRNAs and mRNAs in cancers trade-off can help to discover important findings to identify underlying mechanisms and enlighten more molecular underpinnings of different cancers. We observed that the identified miRNAs-mRNA covered a wide range of known functions, mainly signaling pathways and biosynthesis implicated in colon and lung cancers.

To summarize, conserved miRNAs and TFs like miR-195, miR-145, BTF3, Myc and TP53 with their targets could be considered as hallmark genes for future diagnosis and therapeutic researches where their rules could be confirmed by experiments.
